# On the Design, Fabrication, and Characterization of a Novel Thin-Film Electrode Array for Use in Cochlear Implants

**DOI:** 10.3390/mi15070921

**Published:** 2024-07-17

**Authors:** Gülçin Şefiye Aşkın, Sercan Gökçeli, Bilsay Sümer

**Affiliations:** 1 Department of Nanotechnology and Nanomedicine, Graduate School of Science and Engineering, Hacettepe University, Beytepe, Ankara 06800, Turkey; gulcin.askin@hacettepe.edu.tr; 2 Department of Mechanical Engineering, Graduate School of Science and Engineering, Hacettepe University, Beytepe, Ankara 06800, Turkey; sercan.gokceli@hacettepe.edu.tr

**Keywords:** cochlea, cochlear implant, thin-film electrode array, finite element method, lithography tribology, artificial cochlea

## Abstract

Thin-film electrode arrays (TFEAs) have been developed as an alternative to conventional electrode arrays (CEAs) used in cochlear implants. However, TFEAs produced by microfabrication techniques have not yet been used clinically because their structural and mechanical properties are far from those of CEAs. The aim of this study is to design, fabricate, and investigate the mechanical and tribological behavior and evaluate the performance of different TFEA designs. Finite Element Analysis (FEA) is performed to determine the elastic properties of several designs. A custom-build experimental setup is designed to observe the tribological behavior in different speeds and environments where frictional (lateral) and vertical force (normal force) are measured on a flat surface and within artificial cochlea. According to the FEA results, the maximum stiffness of the CEA is 37.93 mN/mm and 0.363 mN/mm and TFEA-4 has a maximum stiffness of 39.08 mN/mm and 0.306 mN/mm in the longitudinal and transverse axes, respectively. It is shown experimentally that adding a dummy wire to the carrier of the EA enhances both its longitudinal and transverse stiffness, thereby postponing the initiation of dynamic sliding due to the elevated buckling limit. It is also revealed that the type of TFEA support structure affects both normal and frictional forces, as well as the coefficient of friction.

## 1. Introduction

Hearing loss affects individuals of all ages worldwide. Sensorineural hearing loss is one of the most common types and is caused by the absence or damage of hair cells in the inner ear’s cochlea. This can be congenital or due to the destruction of hair cells resulting from disease or ageing. According to the Food and Drug Administration (FDA), a cochlear implant is a Class 3 medical device designed to address hearing loss resulting from damaged hair cells in the cochlea [1,2]. Basically, the cochlear implant is a surgically implanted electronic device and comprises a microphone, a speech processor, a transcutaneous link, an implanted receiver/stimulator, a multi-wire cable, and an electrode array (EA) [2]. 

The most challenging component of the cochlear implant is the electrode array. The electrode array (EA) is a crucial component of the system as it stimulates the auditory nerve to enable hearing. During a typical surgery, the electrode array leaves the implant bed, passes through the facial recess into the middle ear, and enters the cochlea through the round window. Each conductor in the electrode array ends with an electrode surface and this structure can provide artificial hearing by stimulating the auditory nerve.

Cochlear implants (CIs) are manufactured and sold on the international market by a few companies. The main obstacle to the low-cost production of these implants, which are implanted thousands of times a year in medium-sized countries around the world, is the costly electrode array manufacturing process, which requires intensive labor and precision. In a controlled environment, many quality control steps and tests are required to achieve high quality standards in the production process, which involves soldering electrically conductive wires with a diameter of 20–30 µm onto the stimulation electrodes one by one under a microscope with the dexterity of a specialist worker, and micro-injecting a silicon carrier. This significantly increases the unit cost of the cochlear implant. The cost of the implant therefore limits access to cochlear implants around the world.

A CI replaces the process of capturing and amplifying the mechanical energy of sound in the normal hearing mechanism and creates sound perception by directly electrically stimulating the auditory nerve (cochlear nerve). Although the regions that these systems stimulate, and accordingly the stimulation parts and software they use, differ, the other parts and their general functioning logic are very similar. All of these systems end with an electrically conductive metal composite structure called the electrode array (EA). The CEA has traditionally been manufactured using conventional methods, resulting in electrode arrays that range from approximately 16 to 31.5 mm in length, 0.3 mm tip diameter to 0.8 mm base diameter sizes, with up to 24 electrodes, and are available in straight or wavy wire forms, as well as in different EA shapes [3]. In the CEAs, there are electrode surfaces of different shapes with an area of approximately 0.12 mm^2^–1.5 mm^2^ [4] depending on the number of stimulations, electrically conductive wires with a diameter of 20–30 μm, and an elastomer-based polymer as a carrier. The design of various lengths, numbers of electrodes, and physical shapes of wires of EAs depend on diverse individual conditions. Several main factors are different cochlear structures, hearing condition or level, and financial considerations. Today, CIs are accepted as clinically successful [5]. However, the narrow frequency range, inability to distinguish sounds in crowded environments, high cost, and long production process are significant drawbacks of this technology. As a result, researchers have turned their attention to developing thin-film electrode arrays (TFEAs), which could provide a cost-effective alternative to CEAs. 

With the increasing development and diversification of microfabrication and flexible electronic methods in recent years, the feasibility of manufacturing flexible thin-film electrode arrays has increased. Therefore, the TFEA is an attractive option due to its higher number of electrodes, lower cost due to its manufacturing method, and mass production capability. Furthermore, it can provide high-intensity stimulation sites, better performance, and mechanical strength [6]. The design and fabrication of the TFEA use Micro Electro-Mechanical System (MEMS) fabrication techniques. TFEAs have been produced as a solid structure using silicon-based, polymer-based, or hybrid-based materials [6,7,8,9,10,11,12,13,14,15,16,17,18,19,20]. However, silicon-based and hybrid-based electrode arrays lack the flexibility required to move through the cochlea, making them unsuitable for clinical use. Therefore, research has focused on TFEAs with elastomer-based flexible polymer layers in all parts except the electrically conductive layer. Previous studies have frequently employed Parylene as a protective or carrier layer in MEMS manufacturing techniques [6,12,16,17]. However, the modulus of elasticity of this polymer is much higher than that of the elastomer-based polymer commonly used in the CEA, resulting in greater longitudinal and transversal stiffness, which increases the risk of cochlear damage. Long-term fabrication methods, such as RIE, are used to expose the electrode surfaces in MEMS fabrication. Silicone elastomer is used as the carrier layer in addition to Parylene, resulting in a three-dimensional structure [13,14,15]. The polymer thin film is placed flat on the carrier layer in these studies [13,14,15]. It is observed that the film separates from the carrier layer over time [14]. Hence, a TFEA manufactured using MEMS fabrication techniques has not yet found clinical application, primarily due to significant disparities in their structural and mechanical properties compared to CEAs.

The size, shape, and stiffness of the electrode array (EA) are crucial factors for its integration into the cochlea [21,22]. Studies have shown that the stiffness of electrode arrays (EAs) used in cochlear implants is associated with trauma during implementation [21,23,24,25]. It is reported that commonly used TFEAs have drawbacks related to the mechanical properties of their constituent materials, presenting difficulties for their applications in cochlear implants. A low stiffness value is also found to be one of the challenges of EAs and can lead to overbending or folding when the EA comes into contact with the cochlear wall because of high elasticity [15]. The EA must be stiff enough to propagate inside the cochlea, but if it is too stiff, it may cause damage during implementation. Early versions of EAs used single wire electrodes, but more flexible EAs with multiple wires covered with silicone rubber are now commercially available. The bending stiffness and buckling force of one of the conventional electrode arrays (Nucleus straight array) are measured to be 0.39 mN/mm and 4.91 mN, respectively [23]. In a separate study [24], the buckling force of the Nucleus straight array is measured to be 5.10 mN. If the EA has uniform stiffness or if the elasticity modulus of the tip is higher than that of the back parts, the contact pressure or buckling load may increase, potentially damaging the cochlea [24,25]. It has been observed that the contact pressure can be reduced if the EA has gradual hardness or if only the tip is made of a soft material [24]. The studies conducted for the new generation of the TFEA reveal that a carrier is required to move through the cochlea due to the thin film being produced in a two-dimensional plane structure. It has been observed that the carrier component of the EA affects the hardness [12,16]. In [12], a carrier is proposed that surrounds the thin film and has slits at the back to allow the stylet to move forward, making it easier to place the EA in the cochlea. The bending stiffness of the array depends on the depth of the slits in the carrier and the distance between them. In a study by Xu et al., a flat Kapton tape is adhered to the back of the thin film to act as a carrier [16]. The bending stiffness is typically found to be 0.2 mN/mm. Moreover, the electrode array is found to be harder than Johnson’s. However, these studies lack specific design steps aimed at achieving a level of elasticity in the TFEA comparable to that of the CEAs. In addition, the tribological characteristics of the TFEA have not previously been analyzed in preload and speed-controlled experiments.

**Figure 1:**
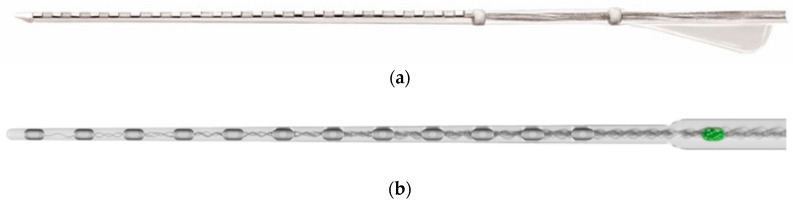
(**a**) Flat wire electrode (Cochlear Corporation [29]); (**b**) Asynchronous waveform wire electrode (MED-EL Medical Electronics [30]).

In recent years, commercially available cochlear implant (CI) electrodes have undergone structural changes in order to prevent tissue damage during CI surgery. As illustrated in Figure 1, the most significant change is the evolution of the metal wires in the electrode array from a straight shape to a wavy shape. Despite the absence of studies in the literature examining this issue in terms of elasticity and bio-tribology, recent artificial model and clinical studies have demonstrated that this structure increases the probability of atraumatic deep implantation into the cochlea without damaging the basilar membrane or other basic neural structures [26,27,28]. The reason for this is that the longitudinal and bending stiffness values of the metal–polymer composite structure are modified by changing the wavelength and the type of wire shape. In this study, the physical dimensions of the TFEA are therefore based on the CEA straight and wave designs. 

In this article, a novel TFEA is designed, fabricated, and characterized. The main objective of this study is to produce a TFEA that is comparable to the conventional electrode arrays that are clinically used and proven. Firstly, a design procedure using the Finite Element Method (FEM) is carried out to understand the mechanical and structural properties of the conventional electrode arrays. A parametric analysis is then carried out to design the TFEA. Furthermore, a novel and custom-build experimental setup is constructed to measure the normal and frictional forces. Finally, a comprehensive tribological characterization of the TFEA is carried out to reveal its frictional properties in both dry and wet conditions, both on a flat glass surface and inside an artificial cochlea.

## 2. Materials and Methods

### 2.1. Physical Dimensions of the TFEA

Three different TFEA designs are proposed in this study. The initial design features a planar thin film with thinly patterned metal electrodes, integrated with an elastomer support. In the subsequent two designs, a dummy wire—either straight or wavy—is incorporated within the support to enhance stiffness along both the longitudinal and transverse directions of the structures.

**Figure 2:**
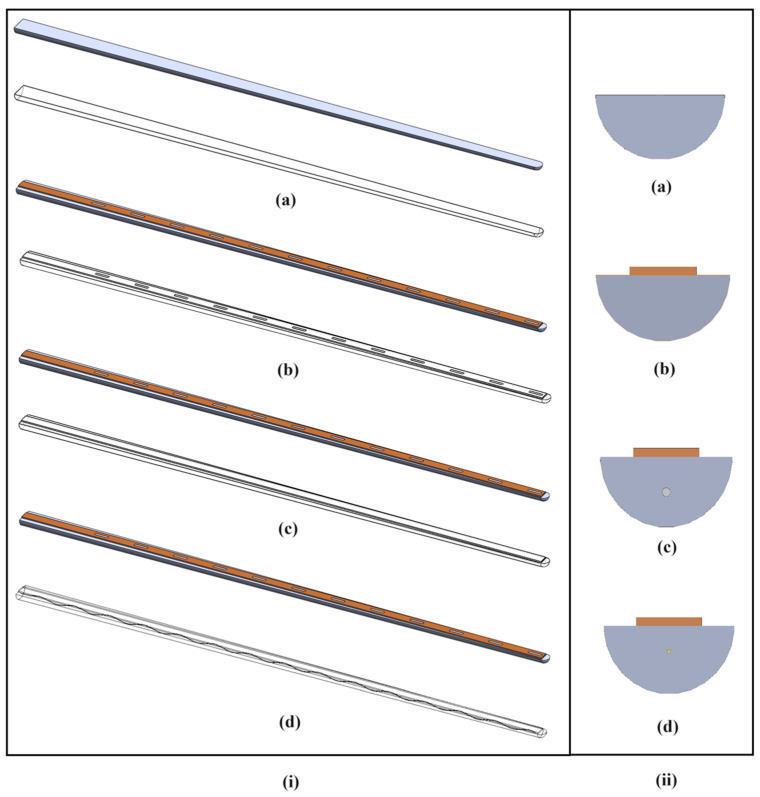
(**i**) Dimetric views; (**ii**) lateral section views; (**a**) TFEA-1; (**b**) TFEA-2; (**c**) TFEA-3; and (**d**) TFEA-4.

The main objective of the design study is to achieve similar elastic properties to the clinically proven CEA for the TFEA. The stages of developing an alternative TFEA design, from the main support component to the final proposed TFEA design stages, are shown progressively in Figure 2. The figure shows the dimensional and cross-sectional views of the designs, with (a), (b), (c), and (d) representing the following: (a) the half-cylindrical cylindrical elastomer support only (TFEA-1), (b) the rectangular thin film mounted on a half-cylindrical elastomer support with an integrated electrode model transitioning to a thin film mounted on a PDMS support (TFEA-2), (c) the structural steel wire-embedded TFEA model (TFEA-3), and (d) the sinusoidal wavy copper wire-embedded TFEA model (TFEA-4). While the substrate of the thin film consists of a polymer film with metal electrodes and lines, there is no layer covering the polymer film, electrodes, and lines. The wires and stimulation electrodes are located in the center.

**Figure 3:**
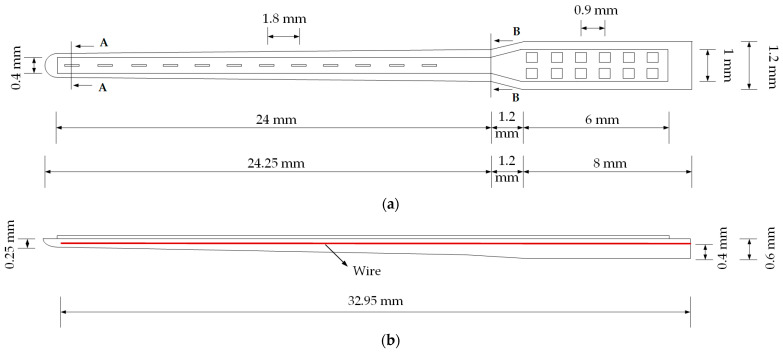

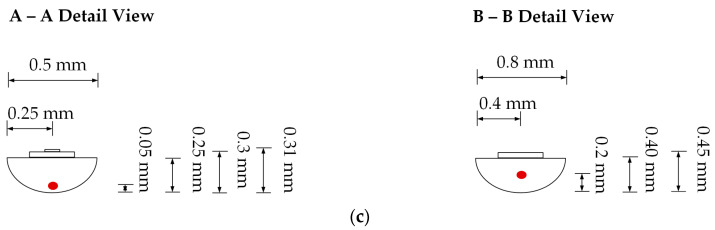
Design of the thin-film electrode array. (**a**) Top; (**b**) side; and (**c**) cross-section view of the thin-film electrode array.

The thin film of the electrode array without a carrier is a planar structure. The part with the stimulation electrodes is 0.4 mm in width and 24 mm in length, while the part with the contact electrode is 1 mm in width and 6 mm in length (Figure 3). The substrate material of the thin film is polyethylene terephthalate (PET) with a 50 µm thickness. The electrode array support (carrier) to be inserted in the cochlea is semi-cylindrical in shape, 24.25 mm long, 0.8 mm wide at the base, and 0.5 mm wide at the tip. These dimensions are in line with conventional electrode arrays [31]. Although there are a variety of conventional CIs, the most common dimensions are selected for simulation purposes and labeled as CEA. CEAs are designed to form a full cylinder. The dimensions of the CEA are 0.5 mm at the tip, 0.8 mm at the base, and 24 mm in length. Embedded platinum corrugated wires have 25 µm diameters, 1.6 mm wavelengths, and 0.2 amplitudes. The wire lengths for the CEA are 3.9, 6.7, 9.5, 12.3, 15.1, 17.9, 20.7, and 23.5 mm. The carrier part (TFEA-1) is integrated with the resulting thin film (TFEA-2). In the third design, the straight stainless-steel dummy wire is located inside the support, which is 50 µm in diameter. In the fourth design, a sinusoidal wavy copper wire with an amplitude of 0.075 mm, a wavelength of 1.6 mm and, a diameter of 20 µm is embedded in the carrier. The lengths of the stainless-steel and copper wires are 32.95 mm. The electrode array on the thin film consists of 12 aluminum stimulation electrodes, each with dimensions of 0.1 × 0.6 × 0.001 mm^3^. The aluminum contact electrodes have dimensions of 0.5 × 0.5 × 0.001 mm^3^. The center-to-center distance between the electrodes is 1.8 mm and the distance between the contact electrodes is 0.9 mm. The line width is 10 µm and the line pitch is 8 µm. Figure 3 shows the top and side views of the TFEA-2, while the cross-section shows the placement between the thin film and the substrate.

### 2.2. Design of the Carrier Using the Finite Element Method

Although the TFEA produced by the microfabrication method is more advantageous than the electrode array developed by the conventional method, the geometry that can be obtained at the end of the microfabrication process is a planar structure. However, the cochlea in which the electrode array is placed is a fluid-filled structure in the form of a spiral and conical three-dimensional shape ending in a gradually decreasing diameter. In order to approximate natural hearing, it is important that the electrode array is placed in the cochlea at the exact depth at which it is intended to enter the cochlea so that all electrode tips can be stimulated. For this reason, the electrode array should have a relatively large diameter proximally and a gradually decreasing diameter in a conical shape towards the tip to ensure complete placement and sufficient stiffness. Therefore, the planar structure that can be obtained by microfabrication methods should be transformed into a three-dimensional (3D) and conical-like shape and structure by designing a supporting structure. Furthermore, achieving the right balance between flexibility and stiffness is essential to facilitate efficient insertion and positioning within the cochlea.

The deflection behavior of the TFEAs is investigated under the condition of large deformation in the longitudinal and transverse directions. The Finite Element Analysis (FEA) is facilitated by using the Static Structural Toolbox within version of ANSYS Mechanical 2023 R1. It is performed by applying the same load in the longitudinal and transverse directions, and the deflection and stiffness values of different designs in the two directions are found. 

**Figure 4:**
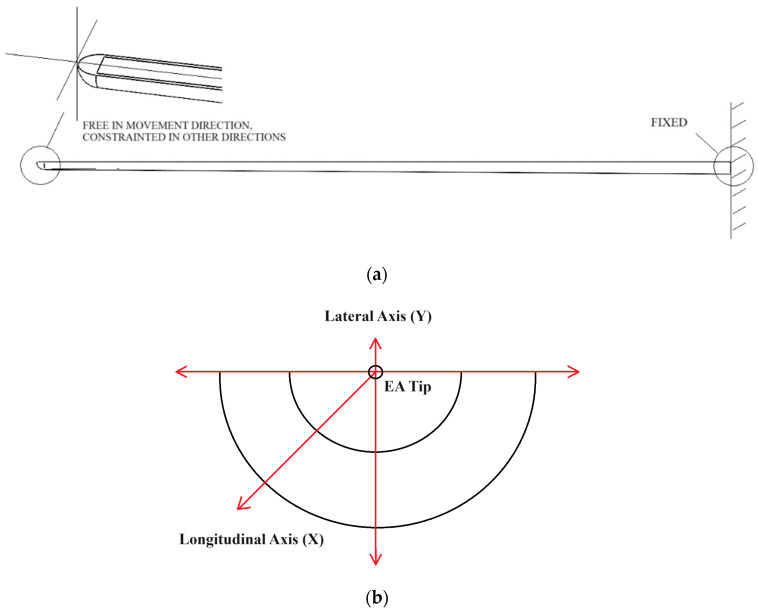
(**a**) Illustration of the boundary conditions of the TFEA; (**b**) illustration of the axis of the TFEA.

Boundary conditions which constrain the advancement of the EA within the cochlear during the surgeon’s operation are listed below. The simplified model of the EA is illustrated in Figure 4a and the defined directions for this study are shown in Figure 4b. The boundary conditions are given as follow:

- The electrode array is fixed at one end. At the tip, the guided boundary condition is applied as the restrained movement can be realized apart from the moving direction of the array.
- Loads of varying magnitude up to 1 mN are applied purely longitudinally (X-axis) or transversely (Y-axis) to the tip of the electrode array.

**Figure 5:**
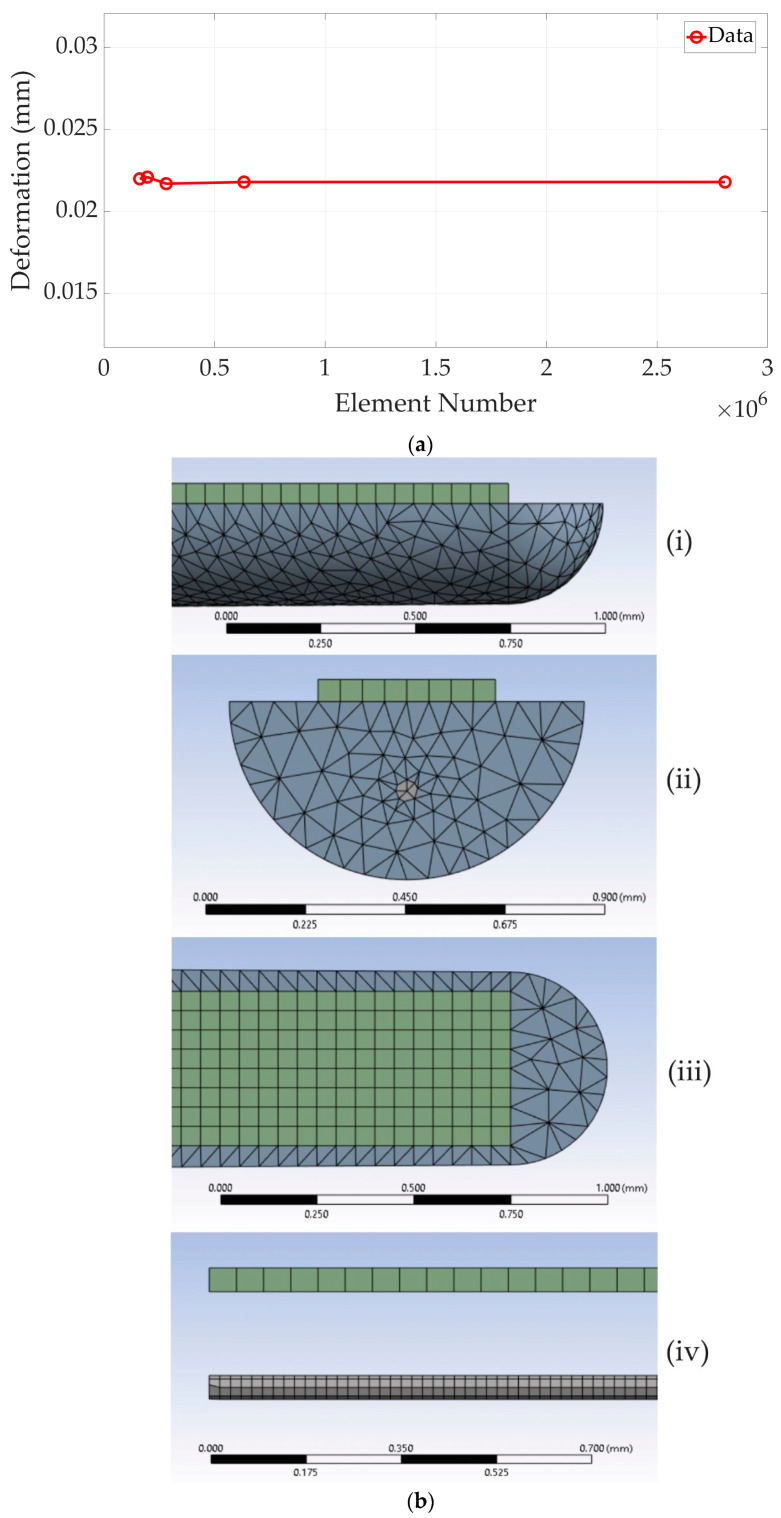
(**a**) A sample mesh convergence analysis; (**b**) mesh structure: (**i**) thin film-PDMS carrier (lateral view); (**ii**) thin film-PDMS carrier (cross-section view); (**iii**) thin film-PDMS carrier (top view); and (**iv**) thin film and wire (lateral view).

Due to their significantly smaller size in comparison to the other components, electrodes are ignored in the FEA process. A shared topology tool is used to ensure accurate and reliable simulation results, as the composite nature of the structure results in drastically changing mechanical properties and dimensions in the contact areas. Conformal meshing can be ensured using the shared topology feature. In this case, different element sizes can also be realized throughout the structure. The mesh convergence analysis given in Figure 5a as a sample is performed for TFEA-3 on the lateral axis by applying a static lateral force on the tip. It shows no progress of accuracy as the number of mesh elements is increased. The first point in the figure corresponds to a 0.05 mm mesh size and 162,021 elements. This value is selected and used in the corresponding analyses. Also, a mesh structure demonstration is given in Figure 5b.

The materials, and their properties, used in this study for the components of the system are given in Table 1. One of the most widely used elastomers for its biocompatibility, polydimethysiloxane (PDMS), is chosen as the carrier. The thin film is made of polyimide, and the wires selected are structural steel and copper metals. All materials are assumed to be elastic and isotropic.

**Figure 6:**
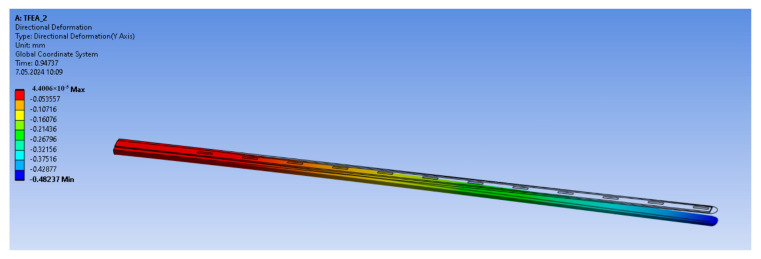
A sample deformation profile of TFEA-2.

A sample Finite Element Model (FEM) and the deflection profile of the thin-film electrode array is shown in Figure 6. Here, the deformed state is seen as a colorful contour and the black skeleton of the structure represents the undeformed state of the EA.

In order to provide a basis for the design process of the proposed TFEA (TFEA-3 or TFEA-4), as an alternative to the CEA, the starting basic concept chosen is a semi-cylindrical support body, as shown in Figure 2a. 

**Figure 7:**
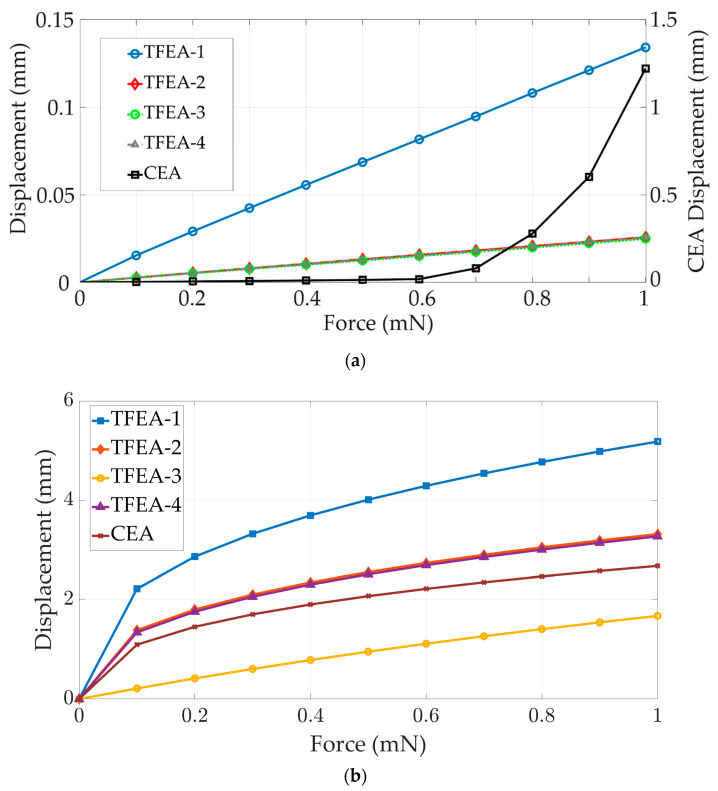
Displacement—force graph: (**a**) longitudinal; (**b**) lateral.

**Figure 8:**
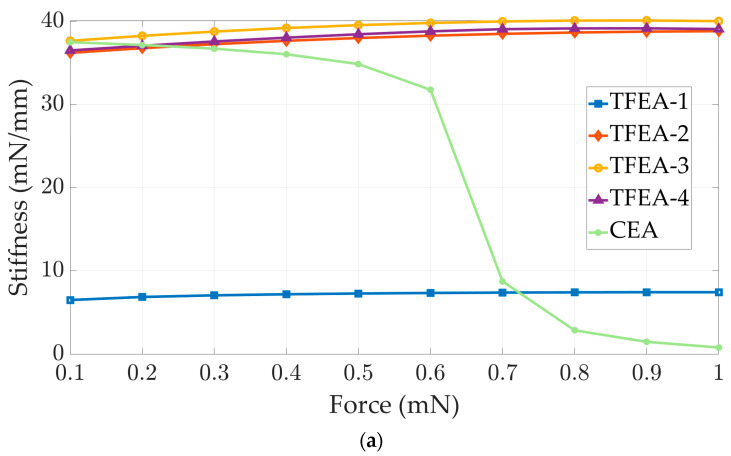

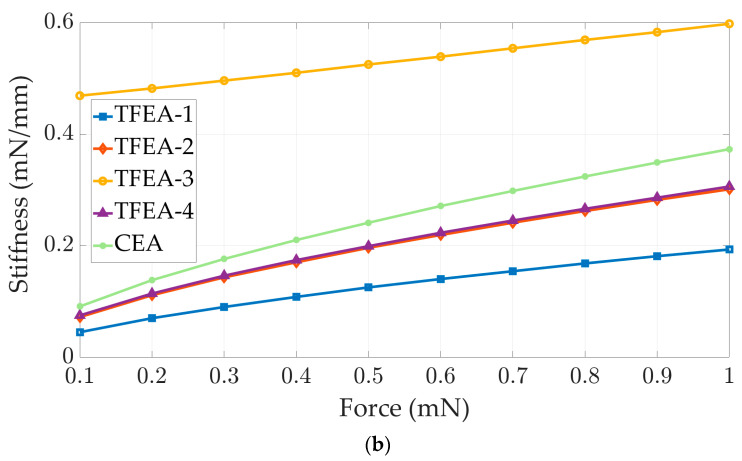
Stiffness—force graph: (**a**) longitudinal; (**b**) lateral.

As expected, TFEA-1 exhibits high flexibility, which can be seen in Figure 7 and Figure 8. It is worthwhile to highlight that multiple y-axes are used in Figure 7 to ensure efficient reviewing of the outcomes, since drastic variation between the ranges of results for different designs is observed. The PDMS carrier cannot resist the load in comparison to other designs and it experiences the highest deflections for all force values and the lowest stiffness in both directions. However, regarding the longitudinal axis, the CEA exhibits highly non-linear deformation behavior above a 0.6 mN magnitude of force and sharply reaches higher deflection levels, as can be seen in Figure 7a. To correlate with the stiffness behavior given in Figure 8a, a sudden decrease in stiffness stems from the fact that the EA experiences buckling and softens under a critical force value, and then load carrying capacity drops rapidly. On the other hand, in respect to the lateral axis, as is shown in Figure 8b, the same behavior is not observed for the CEA. 

In the next step, the thin film integrated with the PDMS carrier (TFEA-2) is investigated for its deflection behavior. A thin film made of polyimide material provides strength to the structure due to its higher Young’s modulus value and the increased cross-sectional area of the structure in comparison to TFEA-1. Because of this, TFEA-2 stiffness values approach those of the CEA in both directions, as shown in Figure 8.

With the aim of enhancing the lateral stiffness of the TFEA-2 structure, the incorporation of novel simple methods and materials into the structure is devised and investigated. For example, TFEA-3 is equipped with a structural-steel rod, acting as a dummy wire embedded in the structure. In this way, the TFEA-3 design can be realized. In this case, the highest stiffness values are achieved in both directions for TFEA-3, as seen in Figure 8. However, an excessive lateral stiffness of the structure in comparison to the CEA is obtained. Even so, the stiffness property depends mainly on geometry and mechanical properties, and the Young’s modulus of structural steel is the dominant factor by increasing its effective value over the whole structure. To overcome this problem, corrugated copper wire with a smaller diameter is embedded into the body of TFEA-2 which results in TFEA-4, shown in Figure 2d. According to the FEA results, TFEA-4 has slightly higher stiffness values than TFEA-2 and is closer to the CEA. With these findings, maintaining rigidity in the longitudinal axis makes TFEA-2 and TFEA-4 favorable to the CEA, which has a non-linear decrease. By this enhancement, it is strongly expected that the possibility of undesired folding inside the cochlear channel for the TFEA designs can be decreased. Even if both can be an alternative to the CEA, the idea of embedding a dummy wire into the body can lead to alterations in the overall mechanical properties of the structure by adding extra wires and changing the geometrical features. Therefore, the TFEA-4 design can provide more functionality and flexibility for usage in practical manner according to several simulations. 

**Figure 9:**
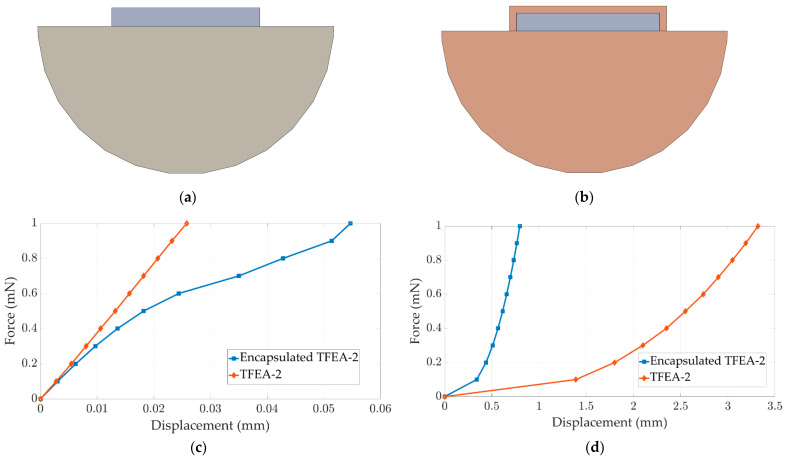
Lateral section views; (**a**) TFEA-2; (**b**) encapsulated TFEA-2; displacement—force graph: (**c**) longitudinal; (**d**) lateral.

While a thin layer of the encapsulation layer is required for clinical application, the encapsulation layer is not applied in this work due to manufacturing limitations. In order to show the effect of the encapsulation layer on the deformation profile, encapsulated TFEA-2 is compared with the original TFEA-2, as shown in Figure 9a,b. The deformations of TFEA-2 and encapsulated TFEA-2 with a 0.02 mm thick PDMS around the thin film under the applied variable force (0 to 1 mN with 0.1 mN increments) are given in Figure 9c,d. Regarding the longitudinal direction, the encapsulated design shows more compliant behavior than TFEA-2 above a magnitude of a 0.6 mN force. On the other hand, in the lateral direction, the stiffer behavior of encapsulated TFEA-2 is observed and strongly depends on the thickness of the encapsulation layer. 

### 2.3. Manufacturing of the Thin-Film Electrode Array

There are two main microfabrication steps that are applied to obtain the TFEA. In the first step, a planar thin-film electrode array is obtained and then a soft molding process is applied to obtain the final structure with a carrier. 

**Figure 10:**
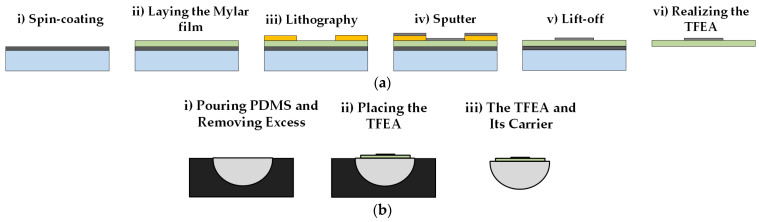

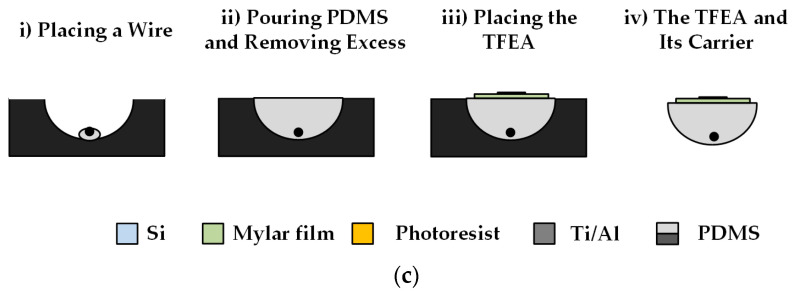
Manufacturing flow chart. Manufacturing scheme of (**a**) thin film; (**b**) carrier; and (**c**) the electrode array with a wire inside the carrier.

Figure 10a shows the fabrication flow diagram. In the first fabrication flow, Mylar film (Dupont, Wilmington, DE, USA) is the preferred substrate for the TFEA. Aluminum (Al) electrodes and electrode leads are fabricated on 50 µm Mylar film using the lift-off process. The first step is to prepare the substrate for the lift-off technique. The Mylar film should adhere to the Si wafer and needs to be fixed during the microfabrication process. This is one of the most challenging processes, as any bubbles or wrinkles between the film and substrate will cause an unsuccessful development process in the lithography step. 

**Figure 11:**
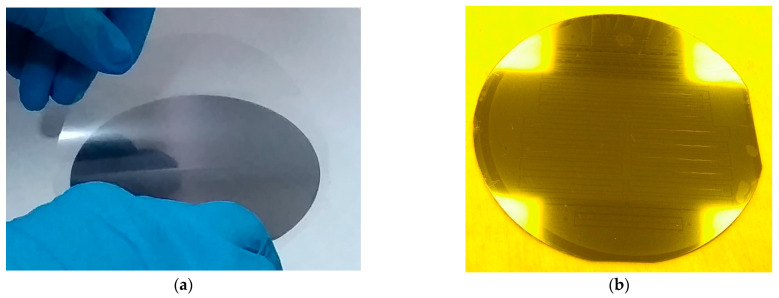

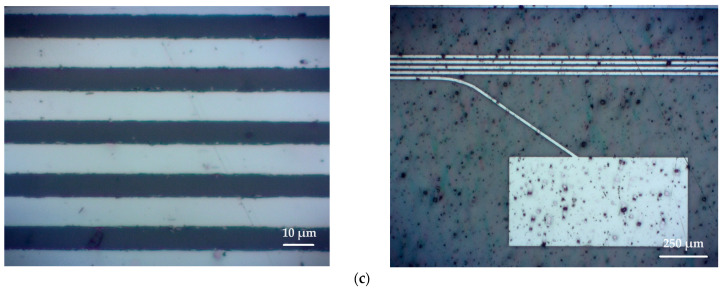
Sample fabrication images: (**a**) example image of the thin film deposition process on PDMS polymer; (**b**) example image of the photoresist after exposure and developer; and (**c**) detailed image of the thin-film electrode lead.

The Mylar film is first cut to a size slightly larger than the Si wafer. The surfaces of the Si wafer and the Mylar film are then cleaned and placed on a hot plate and held at 120 °C for 5 min and 80 °C for 10 min to evaporate the remaining water. Polydimethylsiloxane (PDMS) (Slygard 184, Dow Corning, Midland, MI, USA) is prepared in a ratio of 10:1 and vacuumed to remove air bubbles. The cleaned Si wafer is placed in a custom-built spin-coating apparatus and PDMS is poured onto the wafer. The wafer is spin-coated at 3000 rpm for 50 s to a thickness of approximately 25 µm (Figure 10a(i)). The thin film is carefully placed on top of the Si wafer (Figure 10a(ii) and Figure 11a). Care should be taken to avoid air bubbles during this process, so the wafer is cured slowly at room temperature for 24 h. Here, the use of sudden and elevated temperatures causes air bubbles to form on the surface, wrinkling the thin film and affecting the quality of the subsequent microfabrication process. The next step is to turn the wafer upside down on a clean surface so that the Mylar film remains at the bottom and cut off the excess film where the thin film is ready for lithography.

The second step in the microfabrication process is the fabrication of electrodes and conductors using the lift-off technique. Before working on the prepared substrate, its surface is cleaned again and placed on a heating plate. A positive photoresist (AZ 701 MIR, Microchemicals GmbH, Ulm, Germany) is then spun-coated (~1 µm) for 40 s at 4000 rpm. The wafer is soft-baked on the hot stage at 90 °C for 90 s. After soft-baking, the wafer is placed on the mask aligner (EVG 620 Mask Aligner, EVG Group, Florian am Inn, Austria) and exposure (constant dose, 12.6 s) is performed through the photomask to stimulate the open areas on the photomask. After exposure, the wafer is re-baked on the hot stage at 120 °C for 60 s. To obtain the desired pattern, the wafer is placed in AZ 726 MIF developer (Microchemicals GmbH, Ulm, Germany) for 60 s (Figure 10a(iii)). The wafer is then washed with ionized water and dried with a nitrogen gun. Using sputtering equipment (PVD Handy Twin Sputtering System, VAKSİS R&D and Engineering, Ankara, Turkey), the wafer is first deposited with 10 nm titanium (Ti). Ti, which is preferred due to its structural properties, is used to provide adhesion properties. Al is then deposited at a thickness of 1 µm (Figure 10a(iv)). The wafer is placed in an AZ 100 Remover (Micro-chemicals GmbH, Ulm, Germany) and kept on a hotplate at 60 °C at 700 rpm until the unstimulated photoresist is removed (Figure 10a(v)). The Mylar film is carefully separated from the wafer (Figure 10a(vi)). The TFEAs are cut to a tolerance of 0.02 mm using a precision laser cutter (LS100Ex, Gravotech Inc., Duluth, GA, USA) to separate them.

At least 20 thin films can be produced per wafer by the method of bonding the polymer film to the wafer. In the literature, it is revealed that approximately 8 electrodes will provide good auditory understanding [32].

**Figure 12:**
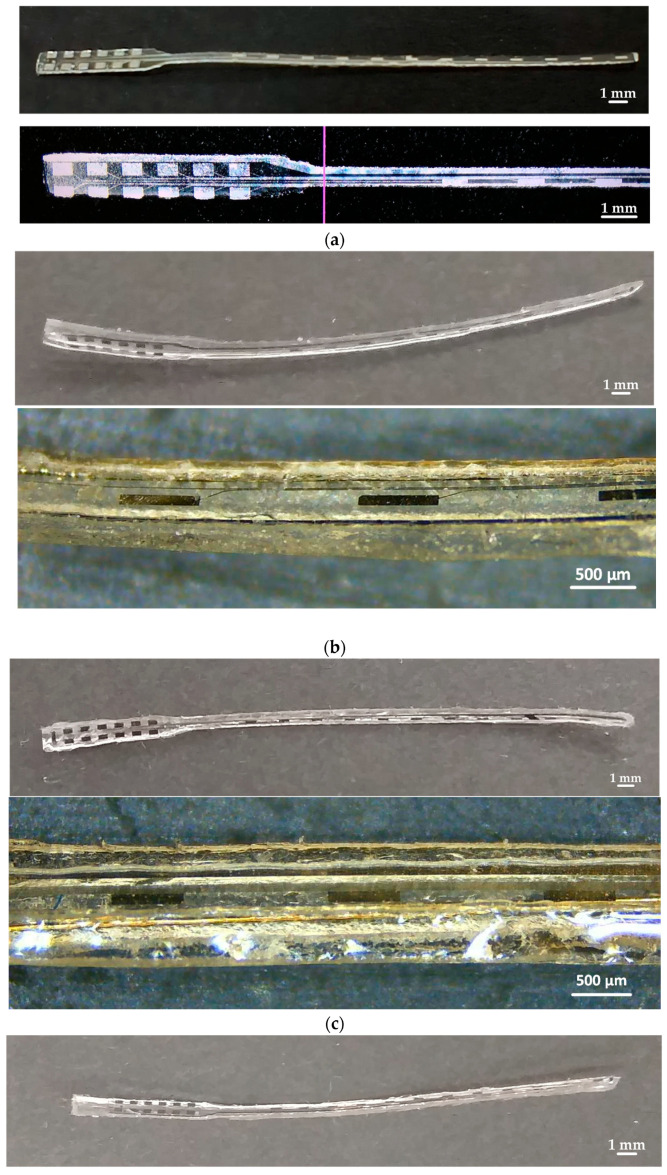

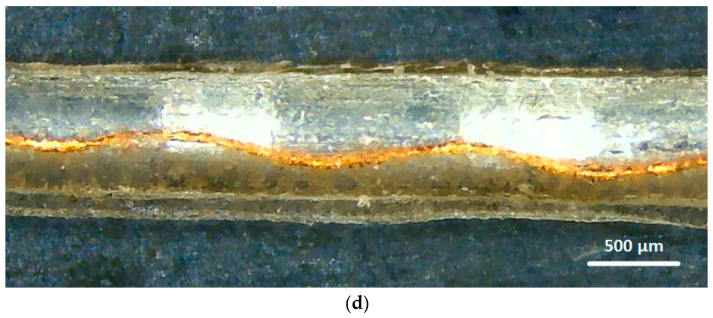
The TFEAs. (**a**) Sample images of thin-film electrode array as a result of laser cutting; images of (**b**) TFEA-2; (**c**) TFEA-3; and (**d**) TFEA-4.

The carrier is attached to the thin film using a soft molding process. Accordingly, PDMS is poured into a semi-cylindrical mold printed on a three-dimensional (3D) printer (Formlabs 3+, Somerville, MA, USA) and the excess is scraped off (Figure 10b). The thin film is applied to the mold using the markers and cured in an oven at 100 °C for 3 h. After solidification, it is removed from the mold using the tweezers. For the dummy wire in the third design, the wire is first placed in the mold and fixed with a very small amount of PDMS and the previous manufacturing flow is followed (Figure 10c). Two different geometries are produced: the single wire inside the support is straight or wavy. While a 50 µm diameter steel wire is used for the straight wire, a 20 µm diameter enameled copper wire is used for the wavy wire. To make the copper wavy wire, two wavy pieces are printed from a 3D printer and the wire is placed between these pieces and manufactured by applying a preload. Figure 12 shows the example of different TFEAs.

### 2.4. Experimental Setups

**Figure 13:**
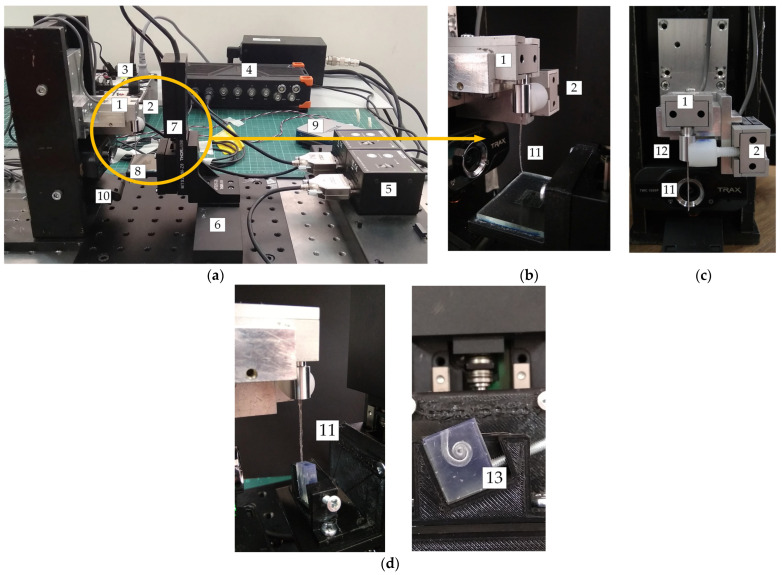
(**a**) Experimental setup: 1—100 g load cell; 2—10 g load cell; 3—signal conditioners; 4—data card; 5—motor controllers; 6—lateral motor; 7—vertical motor; 8—plate; 9—DAQ card; 10—camera; 11—electrode array; 12—holder apparatus; 13—artificial cochlea model; (**b**) close-up view of the EA; (**c**) front view of the EA in the experimental setup; and (**d**) artificial cochlea model experimental setup.

For the tribological characterization of the TFEAs, a novel custom-built test rig is designed. Two different test setups are prepared, referred to as the flat glass surface and an artificial cochlea test setup. In the first experimental setup, the EA is placed on a flat glass surface (Figure 13). The setup contains two load cells (Transducer Techniques, Temecula, CA, USA) mounted perpendicular to each other. While the 100 g load cell is connected to the base of the EA and measures the vertical force (normal force), the 10 g load cell measures the lateral force (friction force) using a device that makes contact with the part to which the EA is attached (Figure 13c). Two motorized translation stages (MTS25-Z8, Thorlabs Inc., Newton, NJ, USA) are used to obtain movement in the vertical and lateral directions. The glass surface is moved vertically towards the EA with the motorized translation stage in the vertical position, providing static contact and generating a vertical force. The motorized translation stage in the horizontal position moves the glass surface in the lateral direction, creating a frictional force between the tip of the EA touching the surface.

In the second experimental setup, the EA is inserted into the artificial cochlea (Figure 13d). The artificial cochlea, designed based on the dimensions found in the literature [33,34], is printed using the clear resin on an SLA 3D printer (Formlabs 3+, Somerville, MA, USA). This setup includes two load cells of the same design as the first test setup and an EA placed next to them. The artificial cochlea is located in a vertical position on the motorized translation stage at an angle of 20 degrees. The motorized translation stage is moved vertically to ensure that the EA is inserted into the artificial cochlea. At the first contact of the tip of the EA within the artificial cochlea, vertical and lateral forces occur and in the meantime the tip is deformed and bends to advance within the cochlea. 

In the first experimental setup, while the vertical motorized translation stage is moved at a constant speed of 0.01 mm/s in all of the experiments, the lateral motorized translation stage is moved at speeds of 0.5 mm/s, 1 mm/s, 1.5 mm/s, and 2 mm/s, respectively. In the second experiment, the vertical motorized translation stage is moved at speeds of 0.5 mm/s, 1 mm/s, 1.5 mm/s, and 2 mm/s, respectively. In the first experimental setup, the speed and preload force of the motorized translation stages are controlled by the LabVIEW program (2018 version), while in the second experimental setup, a dedicated software (Kinesis (1.14.48 version), Thorlabs Inc., Newton, NJ, USA) for the motorized translation stage is used. The force data measured in load cells are recorded in real time on a data acquisition card (Sirius, Dewesoft, Trbovlje, Slovenia). The deformation profile of the EA is monitored with an HD camera during the experiments.

#### 2.4.1. Flat Glass Surface Experiments

Figure 13a–c show the first experimental setup for tribological characterization. In a typical experiment, a vertical motor with a glass substrate is moved at a constant speed towards the stationary EA and the EA comes into contact with the substrate after a certain displacement. After obtaining a predetermined preload by stamping the EA on the substrate, the lateral motor moves a certain distance horizontally from its initial position at a constant speed. It then returns to its initial position at the same speed and the applied force is removed by lifting the vertical motor. The whole process of the EA’s action forms a friction loop. This experiment is carried out in both dry and wet conditions. For the wet environment, a saline solution is used to mimic the body’s natural fluids. Two load cells are connected to the apparatus where the base of the electrode array is located (Figure 13b,c). Due to the sensitive nature of the electrode array and the high immunity of the load cells to noise from their movement, the load cells and EA are preferably stationary in this experimental setup.

#### 2.4.2. Artificial Cochlea Model Experiments

In the second test setup, the EA is inserted in different environments (dry and fluid) within the 3D artificial cochlea model (Figure 13d). The EA and load cells are connected to each other as in the first test setup (Figure 13c). The vertically moving motor is moved at a certain distance and speed to ensure that the EA is inserted within the cochlea. Meanwhile, the vertical and lateral forces measured from the 100 g load cell and 10 g load cell, respectively, are recorded on the data card. The insertion force is the resultant force of vertical and lateral forces.

## 3. Results and Discussion

### 3.1. Flat Glass Surface

**Figure 14:**
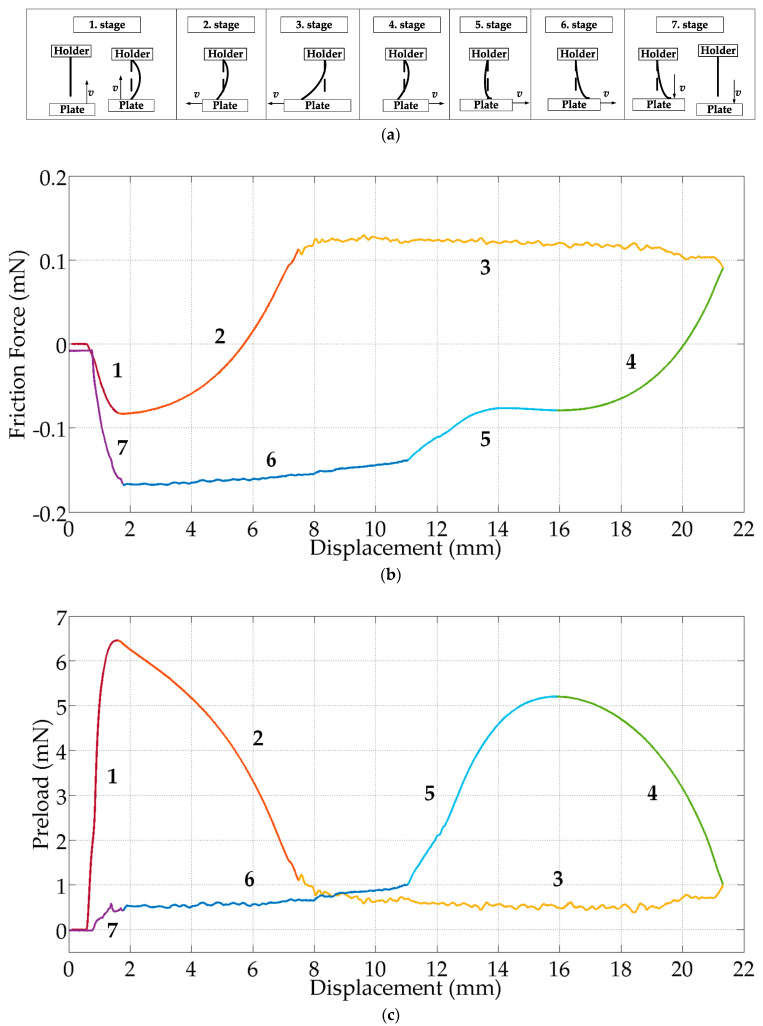
Behavior of TFEA-3 during the experiment when the speed is 1 mm/s under a preload value of *p* = 6.4 mN. (**a**) Illustration pictures of the test showing the behavior of TFEA-3 with the plate during the experiment; (**b**) lateral force—displacement graph; (**c**) vertical force—displacement graph.

Figure 14 shows the typical stages of the friction experiments for the flat surface experiments along with a typical friction loop graph. Friction loops are typical graphs for studying the tribological behavior of the contacting bodies [35]. In the first step of the experiment, the desired preload is obtained by moving the flat plate vertically at a constant speed (*v*) to a certain distance where static contact with the electrode array is achieved. As the electrode array is elastic and thin, it undergoes elastic deformation and buckles immediately after passing the critical buckling load to form a curved shape. The buckling behavior is obvious in stage 1 as there is a negative stiffness in the lateral force (Figure 14b), while there is a positive stiffness in the vertical force (Figure 14) as seen in the experimental results. Positive stiffness corresponds to the force that will return it to its original state against the deformation caused by the force applied in the vertical direction. The negative stiffness can be defined as the positive direction of lateral force is opposite to the buckling direction of the EA. It contributes to the deformation of the TFEA. In the second stage, whenever static contact between the electrode array tip and the flat surface is ensured, the plate is forced to move laterally at a constant speed (*v*). It can be seen that the tip of the electrode array sticks to the plate and the electrode array continues to deform elastically in the lateral direction. Until the dynamic frictional force required to initiate sliding is reached, the vertical force decreases. In stage 3, with the formation of the limiting frictional force, the electrode array slides on the plate and changes from the static to the kinetic frictional stage. In stages 4, 5, and 6, the plate is moved in the opposite direction to return to the starting point of the electrode array. In the fourth case, the movement in the opposite direction begins to show an elastic deformation similar to the second stage, and the tip of the electrode array begins to rotate around itself in the other direction (4th–5th stage). Here, the elastic deformation in the lateral movement disappears and produces a vertical force. When the tip of the electrode array rotates completely in the other direction, its contact with the surface is interrupted for a very short time and a decrease in frictional motion is observed. When it comes into contact with the end plate, the static friction continues its movement. As the movement continues in the opposite direction, a decrease in normal force is observed. In stage 6, the tip of the electrode array reaches the limiting frictional force again and changes from static to kinetic frictional force. In stage 7, the electrode array is released by moving the plate downwards.

In the case that the preload force on the EA is less than the critical buckling force, the frictional force passes from the static phase to the dynamic phase at a certain displacement. In this situation, the derivative of the mechanical energy with respect to displacement is positive. As the electrode arrays are very thin and long structures, the limit of the buckling force value is very low, resulting in immediate buckling behavior. Therefore, in all experiments the preload value immediately exceeds the buckling limits when the EA touches the substrate. Figure 14b shows that the structure is in the negative stiffness state at stage 1 [36]. In the negative stiffness state, the transitions from the static phase to the dynamic phase are greater than in the positive stiffness case; therefore, the transition from static to dynamic sliding is delayed. Here, the force is assisted in deforming the structure, resulting in the easiest motion. However, it also increases the chance of the structure folding in on itself, especially at the tip of the EA. Therefore, the longitudinal stiffness should be increased in order to increase the critical buckling force limit so that higher normal loads can be achieved at the buckling ratio. This would delay the folding of the structure onto itself in the static friction regime, where the dynamic slip region begins immediately.

The friction characteristics of TFEA-2, TFEA-3, and TFEA-4 are investigated at different speed and preloads. Measurements are taken five times for each motion speed and preload and the data are averaged. Whilst TFEA-2 buckles immediately at very low forces, when wire is added to the carrier it becomes stiffer and buckles at higher forces. Therefore, different preloads are applied to TFEA-2, TFEA-3 and TFEA-4 in the experiments. 

**Figure 15:**
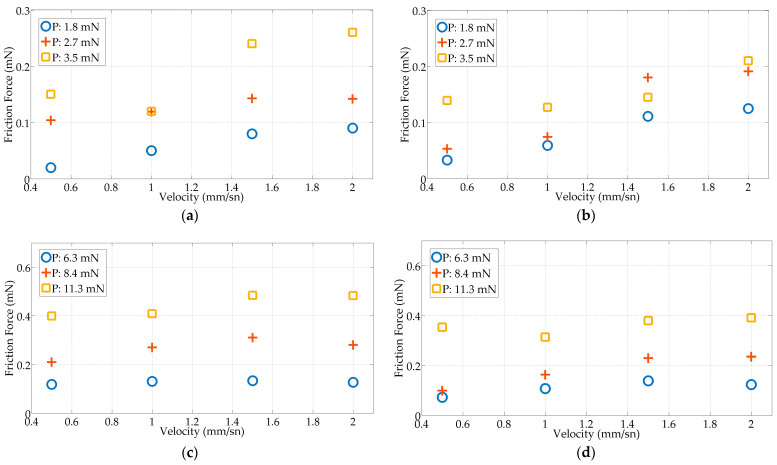

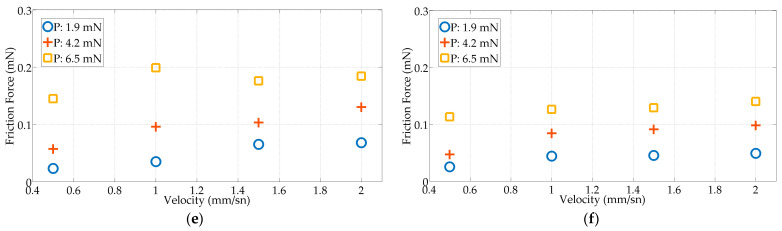
Kinetic friction force–velocity values under three preload values: (**a**) TFEA-2 in dry environment; (**b**) TFEA-2 in wet environment; (**c**) TFEA-3 in dry environment; (**d**) TFEA-3 in wet environment; (**e**) TFEA-4 in dry environment; and (**f**) TFEA-4 in wet environment.

Figure 15 illustrates the effect of frictional force at different preloads (1.8, 2.7, and 3.5 mN for TFEA-2; 6.3, 8.4, and 11.3 mN for TFEA-3; and 1.9, 4.2, and 6.5 mN for TFEA-4) in both dry and wet environments. It can be observed that in both environments, increasing the preload results in higher frictional force values due to the increased surface contact area and the initial state of higher deformation due to the negative stiffness. Additionally, lateral force tends to increase with speed. In general, there is a linear relationship between speed and friction force.

The frictional forces in a wet environment (saline solution) are lower than in a dry environment for the TFEAs because the surface is slippery and it becomes difficult for the tip of the TFEA to stick to the surface. TFEA-4 generates a lower frictional force than TFEA-2 and TFEA-3. 

**Figure 16:**
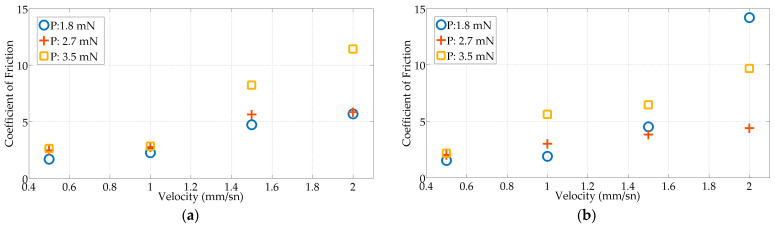

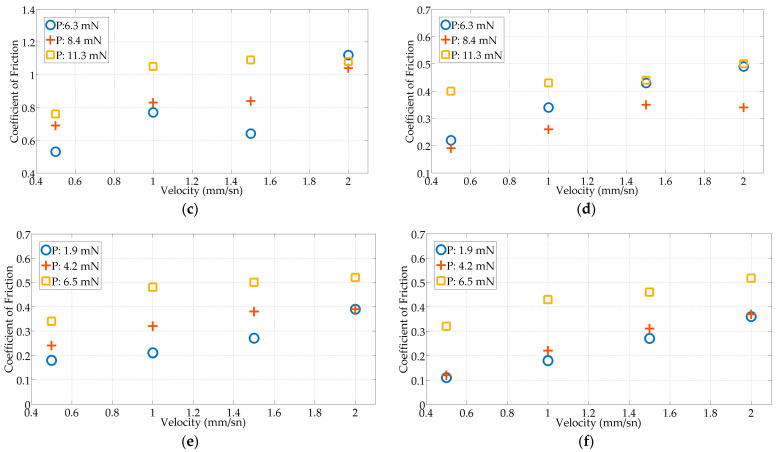
Coefficient of friction–velocity values under three preload values: (**a**) TFEA-2 in dry environment; (**b**) TFEA-2 in wet environment; (**c**) TFEA-3 in dry environment; (**d**) TFEA-3 in wet environment; (**e**) TFEA-4 in dry environment; and (**f**) TFEA-4 in wet environment.

The actual sliding of the EA on the flat surface starts at the beginning of the third stage. Therefore, in this study, the coefficient of friction is calculated by using the kinetic friction force and the preload data in the sliding regime and applying Amonton’s law in the equation as follows:

(1) $${\vec{F}}_{f}=µ{\vec{F}}_{N}$$

where $\vec{F_{f}}$ is the friction force, $\vec{F_{N}}$ is the normal force, and µ is the coefficient of friction. Figure 16 shows the positive average coefficient of friction–speed change graphs of the TFEAs in different environments. Accordingly, the coefficients of friction vary between 1.68 and 11.41 in the dry environment and 1.52 and 14.19 in the wet environment for TFEA-2, 0.53 and 1.12 in the dry environment and 0.19 and 0.50 in the wet environment for TFEA-3, and 0.18 and 0.52 in the dry environment and 0.11 and 0.517 in the wet environment for TFEA-4. TFEA-2 has a higher coefficient of friction than TFEA-3 and TFEA-4. The addition of a dummy wire in the carrier of the EA increases the longitudinal and transverse stiffness, delaying the onset of the dynamic sliding due to the higher buckling limit. The negative stiffness supports the movement of the EA in the transition from static to kinetic friction, thus reducing the coefficient of friction.

Todd et al. [37] measured the coefficient of friction between a Teflon/silicon EA interface with a tribometer device by applying different conditions. The silicone samples used have different geometries (rectangular surface area or spherical geometry) and these samples do not contain wires. They found the dynamic coefficients of friction measured at the Teflon/silicone interface to range from 0.01 to 0.35. Dohr et al. [38] measured the coefficients of friction at different speeds with a friction test device they designed. Accordingly, it was observed that there was a non-linear relationship between friction speed and the coefficient of friction.

### 3.2. Artificial Cochlea

**Figure 17:**
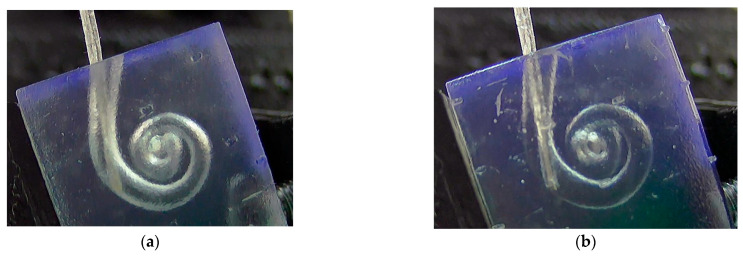
Images of TFEA-3 placed in the artificial cochlea in (**a**) dry and (**b**) wet environments.

In this section, the TFEAs are inserted into the cochlea up to 8 mm at various speeds and in different environments, as shown in Figure 17. Samples are tested five times for each speed, and the resulting data are presented as averages.

**Figure 18:**
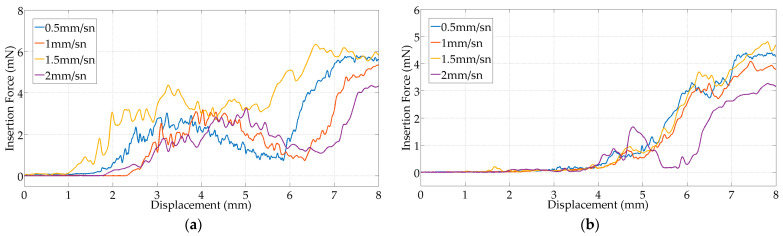

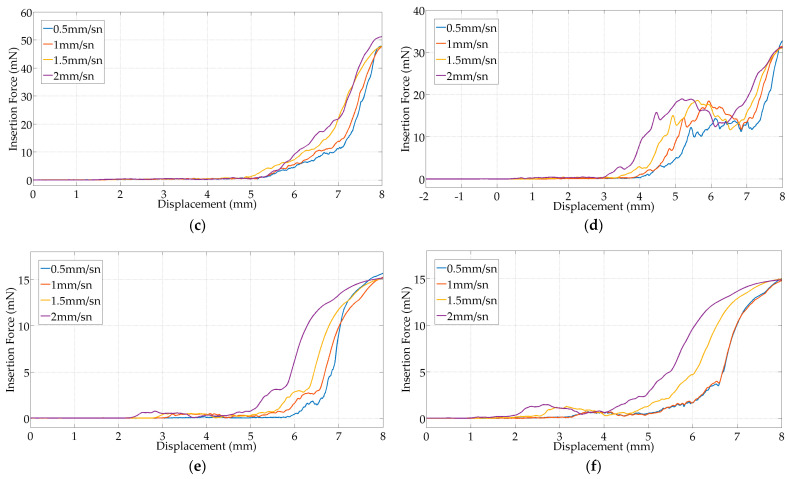
Insertion forces of TFEAs at different speeds. (**a**) TFEA-2 in dry environment; (**b**) TFEA-2 in wet environment; (**c**) TFEA-3 in dry environment; (**d**) TFEA-3 in wet environment; (**e**) TFEA-4 in dry environment; and (**f**) TFEA-4 in wet environment.

Figure 18 shows the insertion forces of the TFEAs at different speeds and environments. As the TFEAs are inserted into the cochlea, it is observed that the insertion force increases in the dry and wet environments. The insertion force of TFEA-3 is about eight times higher in the dry environment and six times higher in the wet environment than that of TFEA-2. It is also approximately three times higher in the dry environment and two times higher in the wet environment than that of TFEA-4 due to its stiffness. TFEA-2 comes out of the mold with a slightly curved shape. As TFEA-2 is inserted into the artificial cochlea, it comes into contact with the inner surface and is further displaced, causing an unstable increase in insertion force. TFEA-3 and TFEA-4 have a flatter shape due to the presence of a wire inside, which makes insertion easier. As can be seen in Figure 18, the different TFEA designs behave differently in terms of insertion speed. When TFEA-2 is inserted into the cochlea, it is observed that as the insertion speed increases in both the dry and wet environments, the insertion force decreases, except for 1.5 mm/s. The effect of speed on the insertion force for TFEA-3 reveals an increase in dry environments and a decrease in wet environments as speed increases. In the wet environment, the insertion force of TFEA-3 first increases with displacement, then gives a decrease in force magnitude after an increase at the end of the movement. In this case, the EA first slides on the surface after a negative stiffness region, which can support movement, and finishes with an increase in resistance to deformation with a positive stiffness. The insertion speed and environment do not significantly affect the insertion force for TFEA-4. Kontronis et al. [39] inserted a conventional electrode array into a Scala tympani model at different insertion speeds (from 10 to 200 mm/min) using the Standard Insertion Technique (SIT) and showed that the insertion force increased with increasing speed. Average insertion forces ranged from 0.09 to 0.85 N and maximum insertion forces ranged from 0.18 to 0.42 N. Rajan et al. [40] also obtained linear results between insertion speed and insertion force. Correspondingly, the negligible influence of speed on insertion force in TFEA-4 renders it more favorable, facilitating the achievement of lower insertion forces compared with TFEA-3. In addition, the TFEA-4 design gives a more repeatable result in terms of its insertion behavior; therefore, it was found to be more robust in terms of its propagation behavior within the cochlea.

The use of lubricant (saline solution) in the artificial cochlea reduces the magnitude of the insertion force. The maximum insertion force is observed in TFEA-3, while the lowest insertion force is observed in TFEA-2. It is observed that the wire integration influences the amplitude of the insertion force. Here, the TFEA-4 design is favorable in terms of the magnitude of the insertion force compared to TFEA-3. Todd et al. [37] found an insertion force of over 50 mN at first contact with the Basal turn area for the SIT in the artificial cochlea for the Nucleus 24 Contour electrode array and this is greater than the insertion force in the TFEAs. The force level of TFEA-2 and TFEA-4 is also found to be in the undamaged limit of the insertion force of the clinically reported CEA force values (29–39 mN) [41]. In addition, the TFEA-4 design has a robust and predictable deformation profile as it moves within the artificial cochlea at all speeds in both environments.

**Figure 19:**
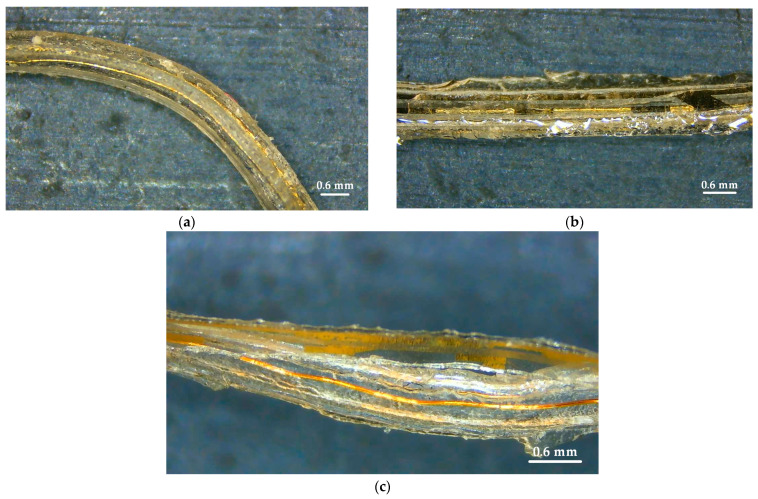
The state of the TFEAs after implantation into the cochlea: (**a**,**b**) TFEA-3 and (**c**) TFEA-4.

During the insertion of the TFEA into the cochlea, it is observed that in one of the seven specimens, the thin film separated from the carrier and damage could occur to the sides of the carrier. However, it should be noted that each sample was used five times in the experiments at different speeds. The typical CIs are only used once. Figure 19 shows the images of two specimens removed after insertion into the cochlea, where there is no separation of the layers.

## 4. Conclusions

This study focuses on the design, fabrication, and analysis of a flexible thin-film electrode array (TFEA) for cochlear implants, which offers several advantages over conventional electrode arrays (CEAs). This study includes the design of TFEAs with three different carrier types to investigate the influence of the carrier component on the tribological performance of the TFEA. These include the carrier parts without a dummy wire (TFEA-2), with a straight steel dummy wire (TFEA-3), and with a corrugated dummy copper wire (TFEA-4). FEAs are carried out to evaluate them in the structural design process. The analyses show that the PDMS structure alone as a carrier could not provide the required stiffness compared to other designs, resulting in high deflection and low stiffness. As the thin film is integrated into the PDMS substrate, the stiffness values of TFEA-2 in both the longitudinal and transverse directions begin to approach those of the CEA in both directions. Further improvements in TFEA-3 give the highest stiffness values but with excessive lateral stiffness. To balance this, TFEA-4 is developed and results show that higher stiffness values can be achieved than with TFEA-2, approaching those of the CEA. Maintaining stiffness in the longitudinal axis and closer stiffness behavior in the transverse axis make TFEA-2 and TFEA-4 favorable alternatives to the CEA. TFEA-4 offers promising functionality and practical flexibility for cochlear implant applications with the incorporation of dummy wires into the flexible design, suggesting potential for further optimization through additional wire embedding and geometric modifications of the wire. Such features of TFEA-4 make it a more practical design regarding FEA results.

TFEAs are manufactured in a two-step process. The first stage involves many microfabrication steps to create patterned electrodes on a thin film. The temporal placement of the thin film on a substrate is a challenging process, as any bubbles or wrinkles between the film and the substrate will result in unsuccessful exposure of the photoresist coated on the thin film. The lift-off technique is effectively used to pattern the electrodes on the thin film. After successful release from the substrate, individual thin-film electrode arrays are cut using a precision laser machine. In the second stage, a soft mold process is used to form the carrier and integrate it with the TFEA without a dummy wire or with a straight or wavy wire. 

The experimental setup is designed and tribology experiments are carried out to investigate the tribological characteristics of the TFEAs. Firstly, a flat glass surface is used as the contact surface to examine the friction characteristics of the TFEA tip between the surface and the contact. A friction loop is used to understand the kinematic and deformation profile of the TFEAs during the tribological tests. It is shown that increasing the longitudinal stiffness is crucial for raising the critical buckling force limit of a structure. This enhancement enables the structure to withstand higher normal loads before buckling, thereby delaying its collapse onto itself. This delay is particularly important in situations where static friction prevails, as it facilitates a gradual transition into the dynamic slip region. Ultimately, this approach strengthens the structure against the buckling and optimizes its performance and stability during dynamic sliding. 

As the preload forces between the flat glass surface and the tip of the TFEA increase, there is a corresponding increase in the frictional force. It is also found that the type of TFEA backing influences both normal and frictional forces, as well as the coefficient of friction. It is found that TFEA-3 has a higher frictional force compared to TFEA-2 and TFEA-4. This difference is attributed to the greater stiffness and adhesion of TFEA-3. In addition, variations in frictional speed lead to changes in frictional force. The TFEAs generally show an increase in frictional force with increasing friction speed.

Secondly, an experiment is carried out by using an artificial cochlea to understand the reaction forces of the TFEAs on the round window, which is the first point of contact when inserted in the cochlea. The experiment is carried out for dry and wet environments as flat glass surface experiments are performed. Under the wet conditions, as expected, insertion force values are lower than under dry conditions. Although TFEA-2 creates the lowest insertion force, it can buckle very easily during insertion into the artificial cochlea. Compared to TFEA-2, TFEA-4 has a higher insertion force, but it is close to the values of the CEAs. 

In the future, the aim is to produce dummy wires within the TFEA that vary in wavelength and amplitude. The effect of these varying wavelengths and amplitudes on the structural and tribological properties of the electrode array will be investigated. Additionally, a cylindrical TFEA with similar dimensions to the CEA will be designed.

## Figures and Tables

**Table 1:** Material Properties.

Material	Density (g/cm^3^)	Young Modulus (MPa)	Poisson’s Ratio
Polydimethylsiloxane (PDMS) (Carrier)	0.965	1.5	0.49
Polyimide (Thin Film)	1.35	3100	0.34
Structural Steel (Wire)	7850	200,000	0.3
Copper (Wire)	8.96	130,000	0.34

## Data Availability

All data generated or analyzed during this study are included in this manuscript. There are no additional data or datasets beyond what is presented in the manuscript.
